# Complexities of Immune Thrombocytopaenia: An Acute Patient With Multi-Pathology

**DOI:** 10.7759/cureus.104058

**Published:** 2026-02-22

**Authors:** Alex Harker

**Affiliations:** 1 General Internal Medicine, Aneurin Bevan University Health Board, Cardiff, GBR

**Keywords:** acute gastrointestinal bleed, clinical bias, gave, immune thrombocytopenia, intravenous immunoglobulins (ivig)

## Abstract

Immune thrombocytopaenia is a condition characterised by isolated thrombocytopaenia, with diagnosis being particularly challenging in cases with concurrent cirrhosis and portal hypertension. This is an unusual case of a gentleman presenting with an acute gastrointestinal bleed on a background of liver cirrhosis with atypical underlying pathology contributing to the bleeding. They were found to have immune thrombocytopaenia and coexistent gastric antral vascular ectasia, which posed unique therapeutic challenges. This case raises the importance for clinicians to maintain a high index of suspicion for multiple pathologies contributing to the presentation of an acutely unwell patient and to be aware of the role clinical bias can play.

## Introduction

Immune thrombocytopaenia (ITP) is a bleeding disorder characterised by isolated thrombocytopaenia (typically <150,000 u/L) [1]. Whilst the pathogenesis of ITP is not fully understood, it is believed to result from the development of an immunoglobulin G autoantibody that targets the structural platelet membrane glycoproteins IIb-IIIa [2]. There are two forms of ITP: primary and secondary. Primary ITP is ITP without an underlying disorder, whereas secondary ITP has an external factor. Potential causes of secondary ITP are drug-related or secondary to other autoimmune conditions like systemic lupus erythematosus [3].

Whilst ITP diagnosis can be challenging, there is a growing number of treatment options to help manage these cases. These options include corticosteroids, intravenous immunoglobulin (IVIG), rituximab, and thrombopoietin receptor agonists (TPO-Ras) [4]. These therapies have been shown to be successful but do carry the risks of negative effects.

Difficulty arises when diagnosing ITP, particularly as there are several mimicking conditions. One such condition is portal hypertension and the subsequent hypersplenism, which results in pooling and sequestration of all corpuscular elements of the blood, predominantly thrombocytes [5].

This case report describes a 71-year-old gentleman who presented with an acute upper gastrointestinal (GI) bleed on a background of cirrhosis secondary to alcohol consumption. Initial laboratory investigation revealed normocytic anaemia and a critically low platelet count of 8,000 u/L. Urgent management was given in the form of red blood cell transfusions and platelet transfusions. This gentleman was also initially treated as a likely variceal bleed and was started on intravenous terlipressin. Despite this treatment, platelets failed to rise significantly, and the clinical picture remained static.

Further investigation involved reviewing previous oesophago-gastro-duodenoscopy (OGD) reports from earlier in the year and previous blood results from an admission in the previous month. This resulted in establishing that in this presentation, there had been a considerable drop in platelets of 178,000 u/L in four weeks and that there were no underlying oesophageal varices. After discussion with haematology, it was decided to administer IVIG to treat ITP. Following administration of IVIG and further red blood cell transfusions, the clinical and biochemical picture improved.

This case highlights the complexity of diagnosing and managing ITP and outlines the impact a degree of clinical bias (assumption of variceal bleed as opposed to an alternative diagnosis) can have. It reinforces the importance of a comprehensive medical review in terms of assessing previous medical investigations and demonstrates the importance of re-evaluation and considering alternate differentials. Early recognition and appropriate treatment in these life-threatening presentations ensure improved patient outcomes and mitigate the added risk of inappropriate medications.

## Case presentation

A 71-year-old Caucasian male presented to the Resus (Emergency Department) after being found on the floor outside a supermarket. History gathered from the next of kin detailed a 24-hour history initially involving haematemesis and then, latterly, melaena. Past medical history included liver cirrhosis secondary to alcohol consumption, a previous right-sided cerebrovascular accident (CVA), minimal motor deficit, hepatic encephalopathy on lactulose and rifaximin, moderate aortic stenosis, and type 2 diabetes (diet controlled). He had not been started on any new medications within the preceding two months.

On review in Resus, vital signs were notable for mild tachycardia at 102 bpm, significant hypotension at 80/43 mmHg, and tachypnoea with a respiratory rate of 23. A Glasgow Blatchford Score (GBS) was calculated as 14. GBS indicates the risk level for a patient with an acute upper GI bleed, with higher scores (e.g., >10) indicating higher risk, subsequently needing prompt inpatient care and intervention [6].

Physical examination revealed poor inspiratory effort, reduced GCS 13/15, a soft, non-tender abdomen, and no obvious skin changes at that time. This gentleman had an episode of melaena whilst being reviewed. Laboratory investigations revealed significant cytopaenias (Table 1) with a haemoglobin of 101 g/L, haematocrit of 0.32 L/L, platelet count of 8,000 u/L, white cell count of 11.3 x 10^9/L, and a prothrombin time (PT) of 13.8 seconds. Renal function tests demonstrated a raised urea of 9.9 mmol/L from a previous level of 3.4 mmol/L and a creatinine slightly below the reference range at 44 umol/L (secondary to his degree of sarcopenia). Liver function tests showed a reduced albumin level of 25 g/L, but other tests were within normal limits.

**Table 1:** Illustrating the trend in the patient’s blood results

	Units	Reference Range	4 Weeks Prior	Day 0 Hour 0	Day 0 Hour 4	Day 1	Day 2	Day 3	Day 4	Day 5	Day 8
White Cell Count	(x10^9/L)	4.0-11.0	4.4	11.3	8.3	6.9	4.6	4.2	3.9	4.9	3.5
Haemoglobin	(g/L)	130-180	123	101	81	98	100	101	108	104	106
Haematocrit	(L/L)	0.40-0.52	0.37	0.32	0.26	0.30	0.31	0.31	0.33	0.33	0.33
Platelets	(x10^9/L)	150-400	186	8	11	22	36	52	82	100	105
Prothrombin Time	(sec)	9.0-12.0	-	13.8	-	-	-	-	12.1	-	11.9
Sodium	(mmol/L)	133-146	138	139	-	136	138	137	143	136	140
Potassium	(mmol/L)	3.5-5.0	3.5	4.7	-	-	3.5	3.4	3.6	3.5	3.8
Urea	(mmol/L)	2.5-7.8	3.4	9.9	-	7.5	3.3	2.1	1.8	<1.8	2.2
Creatinine	(umol/L)	58-110	54	44	-	45	43	40	40	35	42
Bilirubin	(umol/L)	<21	22	16	-	-	19	19	17	16	17
Albumin	(g/L)	35-50	31	25	-	-	21	22	23	22	23
Alkaline Phosphatase	(U/L)	30-130	95	64	-	-	60	64	65	68	67
Alanine Transaminase	(U/L)	<41	13	<9	-	-	14	12	11	12	10

Initial management included transfusion with 2 units of packed red blood cells (pRBCs) and 1 unit of platelets. This gentleman was also started on intravenous terlipressin for a presumed variceal bleed. A blood film was performed and reported as showing true thrombocytopaenia, no red cell fragments, no other features of haemolysis, and no primitive cells.

Upon further review of this gentleman’s medical notes, it was revealed that an OGD was performed three months prior to his presentation, which did not show any evidence of oesophageal varices. It was therefore felt that a variceal bleed was unlikely, and terlipressin was stopped, especially given his background of aortic stenosis.

Given ongoing melaena, OGD was considered but deferred due to the platelet count and significant risk of procedural bleeding. Alongside the transfusion support, he was started on intravenous proton pump inhibitor (PPI) therapy. Post platelet transfusion, there was a minimal improvement in platelet count from 8,000 u/L to 11,000 u/L.

Subsequently, out-of-hours discussions were had with the haematology team regarding the acute platelet count drop. The medical team felt that the acute platelet count drop was unlikely to be secondary to hypersplenism, given his previously normal platelet count four weeks prior. Discussion was had regarding whether this was a disseminated intravascular coagulation (DIC) picture or ITP. DIC is initially a hypercoagulable state that can lead to micro- and macrovascular clotting, followed by haemorrhage as a result of consumption of clotting factors and platelets due to a positive feedback loop [7]. Typically, DIC causes prolongation of aPTT and a reduction in fibrinogen, which this patient did not have, and coupled with the clinical picture, it was decided that ITP was the more likely diagnosis.

There was a considerable lack of secondary causes of ITP elicited from further review, with no recent antibiotic use, a previously negative infectious disease work-up (these were repeated and remained negative), and no autoimmune diagnosis.

This gentleman was given IVIg (Gamunex) at 1 g/kg and closely monitored in a high dependency setting for a further 24 hours. Following IVIg therapy, the platelet count improved to a point where it was deemed that an OGD could be safely performed.

**Figure 1:**
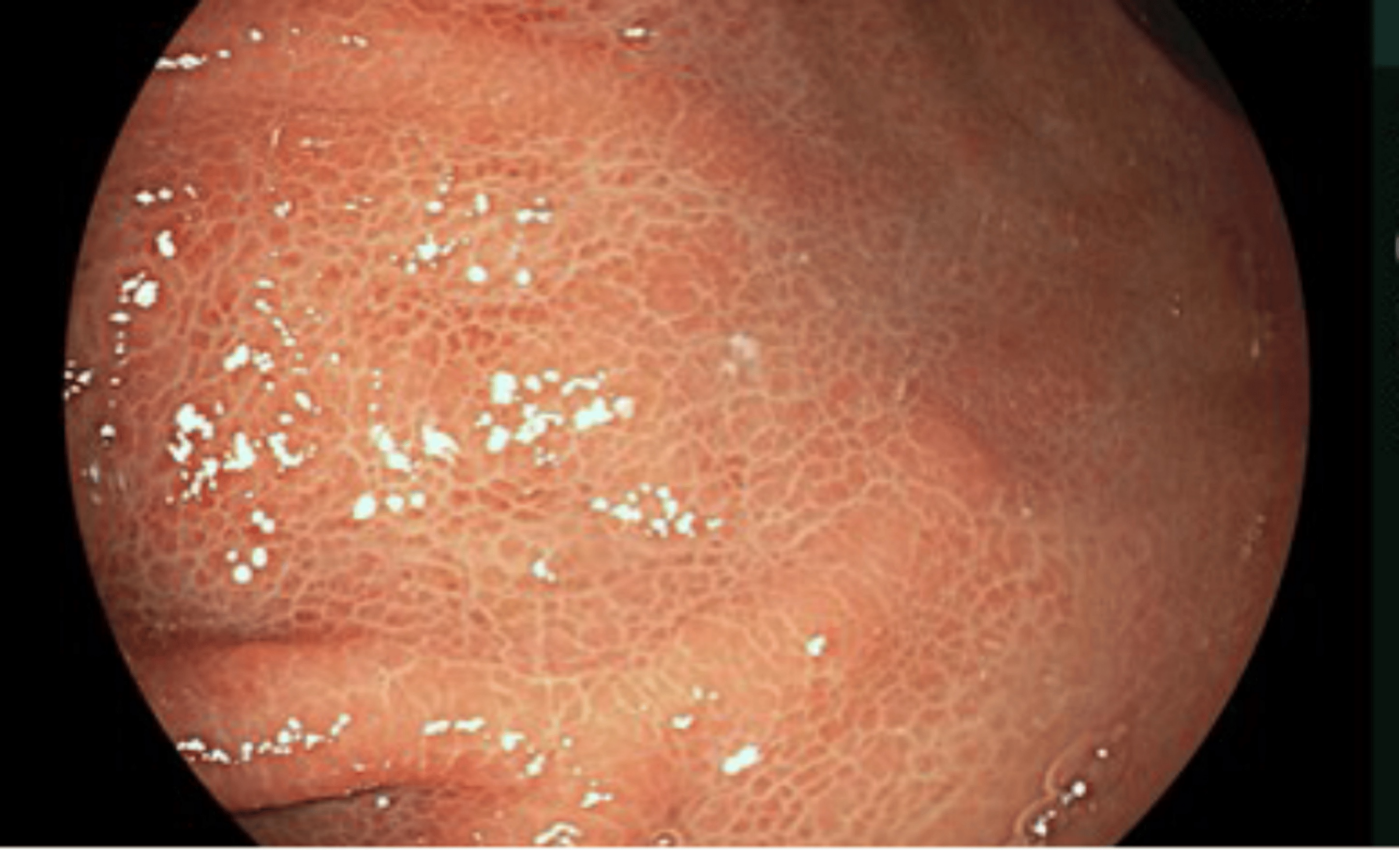
Endoscopic imaging of stomach - middle body Mild portal hypertensive gastropathy

**Figure 2:**
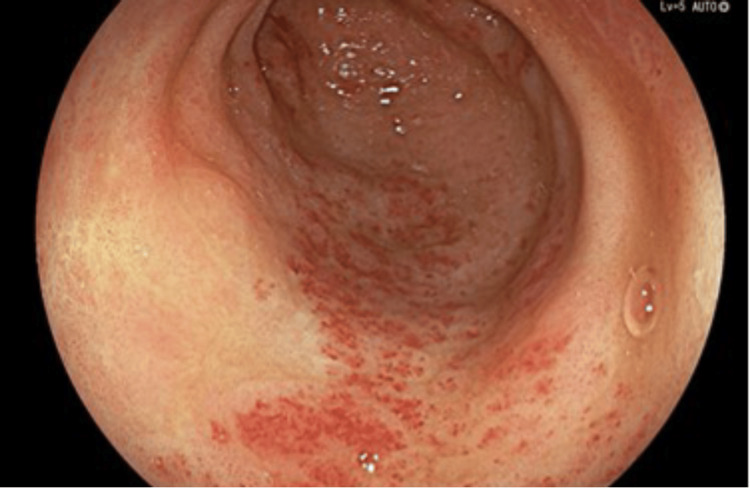
Endoscopic imaging of stomach - antrum Illustrating moderate antral GAVE

**Figure 3:**
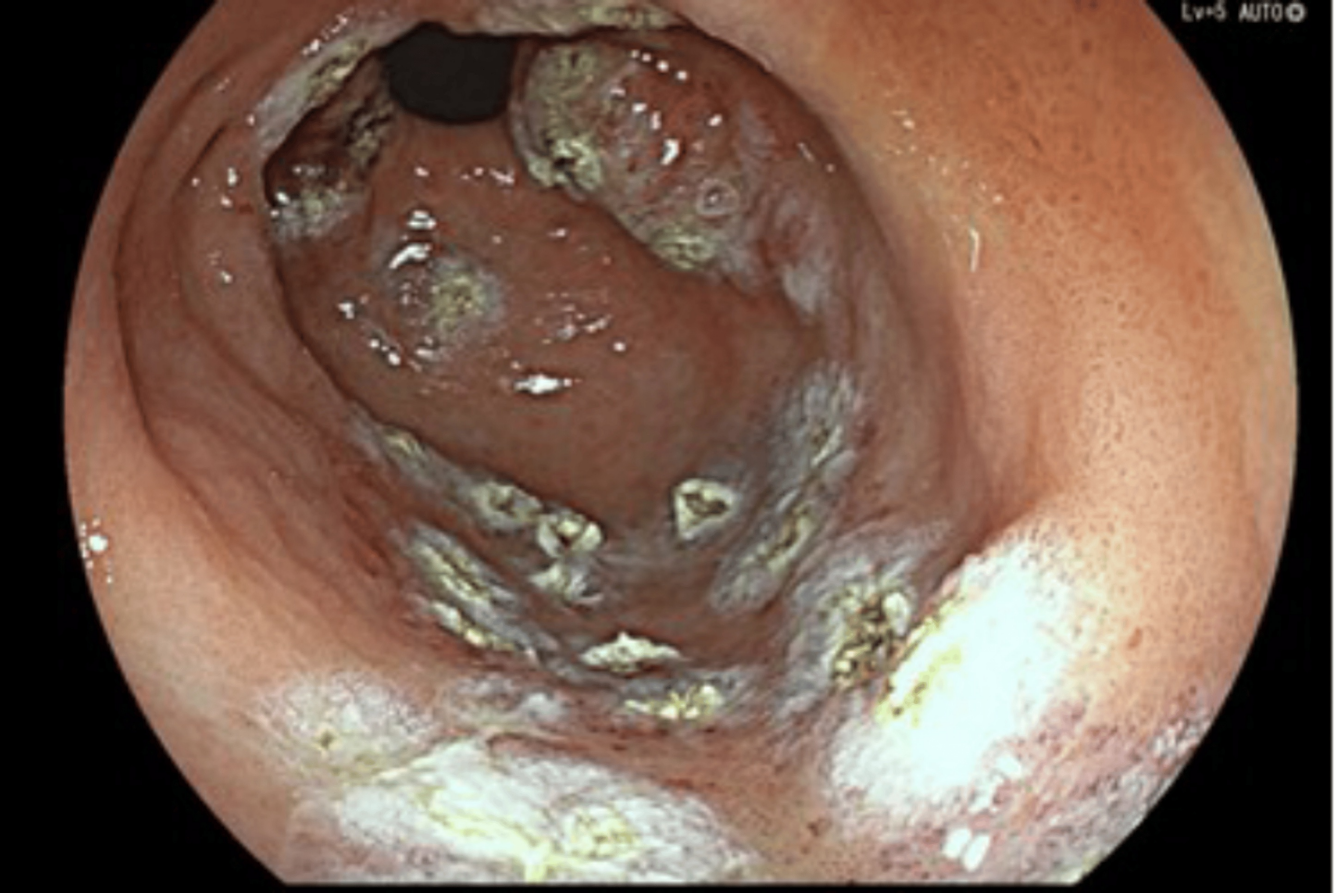
Endoscopic imaging of stomach - antrum (post APC)

An OGD was performed and showed a small sliding hiatal hernia, mild portal hypertensive gastropathy (Figure 1), gastric antral vascular ectasia (GAVE) (Figure 2), which was treated successfully with argon plasma coagulation (APC) at 30 watts (Figure 3), and unremarkable appearances at D1 and D2.

This gentleman was monitored for a further five days in hospital and then discharged with gastroenterology follow-up with close liaison with haematology. He was reviewed in clinic three weeks post-discharge and remained clinically and biochemically stable with no further drop in platelet count.

## Discussion

This case highlights the complexities of diagnosing ITP, particularly in the context of other factors that could not only contribute to thrombocytopaenia but also to GI bleeding.

The incidence of ITP is between 2 and 4/100,000 adults [8], with it being increasingly recognised in older adults over the age of 60. The management of ITP focuses on reducing bleeding risk by increasing platelet count to a haemostatic level. As explored previously, when suspecting ITP, it is important to rule out alternative pathologies, including DIC and other causes, such as drug-induced thrombocytopaenia. In some cases, it may be necessary to perform a bone marrow biopsy to distinguish between ITP and haematological malignancies. In this case, a multidisciplinary team discussion was undertaken, and it was deemed that, given the acuity, this patient’s comorbidities and the clinical picture, a bone marrow biopsy should not be undertaken.

Expanding upon the management of ITP, it is important to instigate supportive measures alongside specific therapy options. These include platelet transfusion, discontinuation of antiplatelet medications, and withholding of starting low-molecular-weight heparin (typically used for deep vein thrombosis prophylaxis). In refractory cases, splenectomy may be considered, but this is less commonly performed due to perioperative risks and complications, including overwhelming post-splenectomy infection.

This case was complicated by the additional diagnosis of GAVE. The pathogenesis of GAVE is unknown; however, it is linked closely with elderly patients and those with renal or hepatic disease. The treatment of GAVE can be divided into three categories: pharmacological, endoluminal, and surgical [9]. From a pharmacological angle, the use of corticosteroids, tranexamic acid, thalidomide, and, more recently, bevacizumab has shown promising results. Endoluminal treatments include sclerotherapy and APC, and surgical options may involve antrectomy. In this case, therapeutic endoscopy was performed once platelets had risen to a safe level, and APC was conducted with good effect.

## Conclusions

Clinicians need to maintain a high index of suspicion for multiple pathologies contributing to the presentation of an acutely unwell patient presenting with a GI bleed. It is important to recognise the role clinical bias plays and to consider alternate diagnoses when the clinical picture does not appropriately fit the primary differential. In these cases, a multidisciplinary team approach is imperative to ensure positive patient outcomes with the early involvement of haematology, gastroenterology, and, where necessary, intensive care medicine.

This patient’s significant bleeding and thrombocytopaenia were secondary to ITP and GAVE and not variceal bleeding as first presumed. Given the acuity of this case, IVIg was administered first as opposed to corticosteroids, and if an appropriate platelet response had not occurred, then rituximab or TPO-Ras would have been considered next line after further consultation with haematology. This case underscores the importance of individualised management plans and treating multiple pathologies concurrently. Careful monitoring and long-term follow-up are essential in these vulnerable patients from both a bleeding perspective and the risk of relapse of ITP potential.
